# Targeted Therapies in Non-Small Cell Lung Cancer—Beyond EGFR and ALK

**DOI:** 10.3390/cancers7020816

**Published:** 2015-05-26

**Authors:** Sacha I. Rothschild

**Affiliations:** Medical Oncology, University Hospital Basel, Petersgraben 4, 4031 Basel, Switzerland; E-Mail: sacha.rothschild@usb.ch; Tel.: +41-61-265-5074; Fax: +41-61-265-5316

**Keywords:** lung cancer, targeted cancer therapy, oncogene, ROS1, c-MET, RET, BRAF, HER2, FGFR1, DDR2

## Abstract

Systemic therapy for non-small cell lung cancer (NSCLC) has undergone a dramatic paradigm shift over the past decade. Advances in our understanding of the underlying biology of NSCLC have revealed distinct molecular subtypes. A substantial proportion of NSCLC depends on oncogenic molecular aberrations (so-called “driver mutations”) for their malignant phenotype. Personalized therapy encompasses the strategy of matching these subtypes with effective targeted therapies. EGFR mutations and ALK translocation are the most effectively targeted oncogenes in NSCLC. EGFR mutations and ALK gene rearrangements are successfully being targeted with specific tyrosine kinase inhibitors. The number of molecular subgroups of NSCLC continues to grow. The scope of this review is to discuss recent data on novel molecular targets as ROS1, BRAF, KRAS, HER2, c-MET, RET, PIK3CA, FGFR1 and DDR2. Thereby the review will focus on therapeutic strategies targeting these aberrations. Moreover, the emerging challenge of acquired resistance to initially effective therapies will be discussed.

## 1. Introduction

Lung cancer is the leading cause of cancer-associated death worldwide [1]. Lung cancer is traditionally classified into non-small cell lung cancer (NSCLC), small cell lung cancer (SCLC), and carcinoid. NSCLC accounts for approximately 80% of all lung cancers and is further subtyped into adenocarcinoma, squamous cell carcinoma, and large cell carcinoma [2]. This purely morphological taxonomy has been challenged in the past decade as it has been recognized that somatic oncogenetic alterations can further molecularly subdivide these NSCLC subtypes. Genotype-driven therapy (“targeted therapy”) is nowadays standard of care for a significant subgroup of NSCLC patients with advanced/metastatic disease.

Genotype-driven treatment with rationally targeted therapies has led to unprecedented outcome improvements. Historically, the estimated median overall survival (OS) for patients with advanced/metastatic NSCLC (stage IV) has been 10 to 12 months. The discovery of activating epidermal growth factor receptor (EGFR) mutations as predictors of a response to EGFR tyrosine kinase inhibitor (TKI) therapy profoundly changed the therapeutic landscape of lung adenocarcinoma [3,4]. More recently, targeted therapies directed at NSCLC harboring anaplastic lymphoma kinase (ALK) fusions and ROS1 fusions have produced similar results in terms of overall response rate (ORR) and progression-free survival (PFS) [5,6]. A recently published analysis showed a significant survival improvement for patients with an oncogenic driver undergoing targeted therapy with a median OS for these patients of more than three years [7]. In addition to EGFR, ALK, and ROS1, there are several other molecular abnormalities that could potentially be treated with drugs already approved for other malignancies or with investigational agents [8].

This review aims at providing an overview on genomic aberrations beyond the well-known and established EGFR mutations and ALK rearrangements. The current evidence on targeted therapies for each aberration, its role as predictive marker, ongoing clinical trials and potential mechanisms of resistance towards targeted therapies will be discussed.

## 2. ROS1

The c-ros oncogene 1 (ROS1) encodes a tyrosine kinase receptor from the insulin receptor family. A rearrangement of ROS1 has initially been described in glioblastoma [9,10,11,12]. In 2007 ROS1 rearrangement was found in NSCLC cell lines and primary tumors [13]. ROS1 fusion partners include SLC34A2, CD74, TPM3, SDC4, EZR, LRIG3, KDELR2, and CCDC6 [14]. A ROS1 rearrangement has been described in 0.7%–1.7% of NSCLC patients [14,15,16]. Similar to previously described oncogenic aberrations in lung cancer, ROS1 translocation is predominantly found in younger patients with adenocarcinoma histology who are never or former light smokers. However, ROS1 translocations have also been described in squamous cell histology [16]. Crizotinib, a potent inhibitor of ALK and MET has also shown activity against ROS1-rearranged NSCLC [15]. An expansion cohort of the phase I trial of crizotinib (PROFILE 1001) included 50 patients with ROS1 translocation [17]. An overall response rate (ORR) of 72% has been reported including three patients with a complete remission. The median duration of response was 17.6 months and the median progression-free survival was 19.2 months with 25 patients still in follow-up for progression when the results have been reported. In a European Cohort (EUROS1) 31 ROS1-positive NSCLC patients have been treated with crizotinib [18]. Median age was 50.5 years, 64.5% of patients were women, and 67.7% were never-smokers. The overall response rate for crizotinib therapy was 80% and the disease control rate was 86.7%. The median PFS in this retrospective analysis was 9.1 months. This cohort study confirms the findings from the prospective trial that crizotinib is a highly active therapeutic option in NSCLC patients harboring a ROS1 rearrangement. Other agents are currently investigated for ROS1-positive lung cancer patients including foretinib, ceritinib, AP26113, PF-06463922 as well as HSP90 inhibitors.

As shown in other molecular NSCLC subgroups treated with tyrosine kinase inhibitors (TKIs), acquired resistance is inevitable and various mechanisms for different molecular alterations have been described. Mechanism of acquired resistance to crizotinib was partly mediated by the ROS1 G2032R mutation in a patients with metastatic adenocarcinoma harboring CD74-ROS1 fusion [19] or EGFR pathway activation in a patient with NSCLC harboring SDC4-ROS1 fusion [20]. Recently, novel ROS1 mutations in lung cancer cell lines as well as epithelial-to-mesenchymal transition have been described to confer resistance to crizotinib [21]. In a preclinical model, the c-MET/RET/VEGFR inhibitor cabozantinib has shown activity in acquired ROS1 inhibitor-resistant mutations [22].

Currently, novel TKIs with activity against ROS1 are under clinical investigation. AP26113 is a small molecule with activity against ALK and ROS1 also inhibiting ROS1-positive cell lines with a ROS1 resistance mutation [23]. Foretinib has shown preclinical activity in cell lines harboring ROS1 translocations and also in cell lines with the G2032R resistance mutation [24]. PF-06463922 is another potent and selective ALK/ROS1 inhibitor showing efficacy in a preclinical model even in cell lines resistant to crizotinib [25]. Another approach currently investigated in crizotinib resistant patients harboring ALK or ROS1 translocations is the combination of ALK/ROS1 and heat shock protein 90 (HSP90) inhibitors [26].

## 3. BRAF

BRAF (v-Raf murine sarcoma viral oncogene homolog B) is a serine-threonine kinase belonging to the RAF kinase family lying downstream of KRAS and directly interacting with the MEK-ERK signaling cascade. BRAF mutations have initially been described in malignant melanoma where 40%–60% of tumors harbor an activating V600E BRAF mutation [27]. Subsequently, BRAF mutations have also been detected in colorectal cancer, papillary thyroid cancer and other solid tumors [27,28,29]. BRAF targeting TKIs (dabrafenib, vemurafenib) are approved for BRAF mutated malignant melanoma based on pivotal phase III trials [30,31]. In lung adenocarcinoma BRAF mutations are found in 1%–5%, half of them harboring the classical V600E mutation [32,33]. Other mutations occur within exons 11 and 15 [33]. BRAF V600E mutations are associated with light/never smoker status, micropapillary histology and occur more frequently in female patients. In contrary, non-V600E mutations are more frequent in former or current smokers and are associated with poorer outcome [33,34].

The BRAF inhibitor dabrafenib is currently tested in a phase II trial. Preliminary results from 17 patients harboring a V600E mutation demonstrate a durable partial response in 54% [35]. Responses for vemurafenib have been demonstrated in several case reports [36,37,38,39]. Preclinical data suggest resistance in non-V600E BRAF mutant melanoma cell lines [40]. This finding is supported by a recent case report showing a rapid tumor progression in a patient with a BRAF G469L mutation undergoing therapy with vemurafenib [41]. Recently, response to dabrafenib in a patients progressing on vemurafenib and docetaxel has been shown [42].

Preclinical data suggests that BRAF activating mutations may predict sensitivity to inhibition of MEK which is supported by clinical response seen with MEK TKIs in BRAF mutated melanoma [43,44]. Furthermore, synergistic activity for the combination of BRAF- and MEK-inhibition has been demonstrated in another preclinical model [45]. Ongoing trials in BRAF mutated lung adenocarcinoma investigate BRAF-, MEK- and AKT-inhibitors.

## 4. KRAS

KRAS belongs to the RAS family of oncogenes together with HRAS and NRAS. KRAS is one of the first characterized oncogenes [46]. KRAS mutations are detected in approximately 20%–25% of lung adenocarcinoma and 4% of lung squamous cell carcinoma [7,47]. Contrary to most other oncogenic driver mutations, KRAS is more often found in smokers and is detected at lower frequency in East Asian patient cohorts [48,49]. Mutations in KRAS are usually mutually exclusive with other oncogenic driver aberrations including EGFR, BRAF, HER2 mutations and ALK and ROS1 rearrangements [47]. KRAS mutations in NSCLC most often occur in codons 12 or 13 and with a lower frequency in codon 61 [7]. HRAS and NRAS mutations are very uncommon in NSCLC [7]. The prognostic as well as predictive role of KRAS mutations is still controversial. In early stage lung cancer, KRAS mutations are neither of prognostic relevance nor are they predictive for the use of adjuvant chemotherapy [50]. However, a large meta-analysis detected a negative prognostic impact of RAS mutations in NSCLC, especially in adenocarcinoma patients [51]. In metastatic NSCLC KRAS mutations did not predict response to standard chemotherapy [52,53]. However, KRAS mutations seem to negatively predict response to EGFR TKIs [54,55,56].

Although various attempts inhibiting KRAS have been made, there is no established therapy for this large patient subpopulation. Initially, direct targeting of mutant KRAS with farnesyl transferase inhibitors has been investigated with only minimal clinical activity [57]. The most promising approach for KRAS mutant lung cancer seems to be the inhibition of MEK in combination with conventional chemotherapy. In a phase II trial in 87 previously treated KRAS mutant NSCLC patients the addition of the MEK1/2 inhibitor selumetinib to docetaxel improved ORR (37% *vs.* 0%, *p* < 0.0001) and median PFS (5.3 *vs.* 2.1 months, HR 0.58, *p* = 0.014) with a trend towards longer overall survival (9.4 *vs.* 5.2 months, HR 0.80, *p* = 0.21) [58]. The combination showed significantly higher grade 3/4 toxicity (45% *vs.* 4%) with neutropenia, febrile neutropenia and pneumonia being the most frequent grade 3/4 toxicities. Currently, a phase III trial with selumetinib is ongoing (SELECT-1, NCT01933932). Trametinib is another inhibitor of MEK showing activity in KRAS mutant NSCLC patients in combination with docetaxel or pemetrexed [59]. However, trametinib single agent could not improve the outcome of KRAS mutant patients compared to docetaxel in the second-line setting [60]. Several clinical trials are currently investigating selumetinib and trametinib in combination with chemotherapeutic agents. Other therapeutic approaches for KRAS mutant lung cancer are the inhibition of other downstream signaling pathways as PI3K and focal adhesion kinase (FAK) [61,62]. Furthermore, direct KRAS G12C inhibitors have shown activity in preclinical models [63,64]. Also the combination of two targeted agents interacting with the Ras/Raf/MEK/ERK pathway might be a future option for these patients, e.g., selumetinib plus the AKT inhibitor MK-2206.

NRAS mutations have been found in 1% of NSCLC, more commonly in adenocarcinoma patients with a smoking history. In preclinical models, these tumors appear sensitive to MEK inhibitor treatment [65].

## 5. HER2

Human epidermal growth factor receptor 2 (HER2) is a member of the ERBB receptor tyrosine kinase family. It is activated by homo- or heterodimerization. In breast cancer, HER2 amplification occurs in about 20% of patients and is a predictive marker for anti-HER2 antibodies and TKIs [66,67,68]. In NSCLC, amplification of HER2 detected by FISH is found in 2%–4% of NSCLC patients. HER2 overexpression by immunohistochemistry is detected in 13%–20% of NSCLC samples, although strong expression is only found in 2%–4% [69,70]. HER2 aberrations are more prevalent in adenocarcinoma patients and HER2 amplification is a negative prognostic marker as shown in a recent meta-analysis [71]. About 1%–2% of adenocarcinoma patients harbor mutations in the exon 20 of HER2 [72,73,74]. These mutations are not clearly associated with HER2 amplification. Anti-HER2 therapies have not shown efficacy in HER2-amplified NSCLC [75,76,77]. However, in a European cohort study HER2 mutation positive adenocarcinoma has been shown to be responsive to HER2-targeted therapies with an ORR of 50% and a disease control rate of 83% [78]. In patients treated with chemotherapy in combination with an anti-HER2 therapy the disease control rate was 93%. The median PFS in this cohort was 5.1 months. Afatinib, a TKI with activity against ERBB family members is approved for EGFR mutation positive adenocarcinoma and has shown clinical activity in lung cancer patients harboring a HER2 mutation even after failure of other EGFR- or HER2-targeting therapies [78,79]. Neratinib is an irreversible pan-HER inhibitor showing clinical activity in HER2-mutated NSCLC patients in a phase I trial combined with the mTOR inhibitor temsirolimus [80]. Currently, several clinical trials are investigating the role of HER2-directed antibodies (trastuzumab, pertuzumab) as well as HER2-targeting TKIs (afatinib, dacomitinib and neratinib).

## 6. c-MET

Mesenchymal-epidermal transition (MET) is a receptor tyrosine kinase, which undergoes homodimerization by binding its ligand, hepatocyte growth factor (HGF). Homodimerization and autophosphorylation of MET leads to the activation of various intracellular signaling pathways including RAS-RAF-MAPK and PI3K-AKT-mTOR [81]. In lung cancer, MET mutations are rarely detected [82], amplifications are found in around 2%–5% of NSCLC, predominantly in adenocarcinoma [83,84]. However, in a Japanese cohort an amplification rate of 21% has been described [81]. MET overexpression occurs in more than 25% of NSCLC and is associated with poor prognosis [85,86]. MET amplification has been described as one potential mechanism of resistance towards EGFR inhibition in EGFR mutant lung adenocarcinoma [87,88].

Various approaches targeting MET have been investigated. However, most of the trial did not select for MET-specific patient cohorts but investigated the role of MET inhibition in unselected lung cancer patients. Onartuzumab is a monovalent, monoclonal antibody against HGFR. In a randomized phase II trial onartuzumab was investigated as second- or third-line treatment option in combination with erlotinib and compared to erlotinib in combination with placebo [89]. There was no difference in outcome in the whole patient population of 137 patients. However, 66 patients with immunohistochemistry positive (2+ or 3+) showed significantly longer PFS (median 2.9 *vs.* 1.5 months, HR 0.53, *p* = 0.04) and OS (median 12.6 *vs.* 3.8 months, HR 0.37, *p* = 0.002) when treated with onartuzumab. In a phase III trial 499 previously treated MET-positive NSCLC patients were randomized between erlotinib plus placebo and erlotinib plus onartuzumab [90]. This trial could not confirm the results from the previous phase II trial. An independent data review committee recommended to stop the trial for futility, as the addition of onartuzumab to erlotinib did not improve OS (median 6.8 *vs.* 9.1 months, HR 1.27, *p* = 0.068), PFS (median 2.7 *vs.* 2.6 months, HR 0.99, *p* = 0.92), or ORR (8.4% *vs.* 9.6%, *p* = 0.63). Based on these negative results, the development of onartuzumab for NSCLC was stopped. Ficlatuzumab and rilotumumab are two other MET antibodies showing clinical activity in phase I trials and are currently investigated in NSCLC patients [91,92].

Tivantinib is a multi-kinase TKI with high affinity for the inactive kinase domain of MET. In a phase II trial 167 previously treated but EGFR TKI naïve NSCLC patients were randomized to erlotinib plus placebo or erlotinib plus tivantinib [93]. The trial arms were balanced with regard to MET amplification. The combination of erlotinib plus tivantinib did not improve the primary endpoint PFS (median 2.3 *vs.* 3.8 months, HR 0.81, *p* = 0.24). Also within the MET-positive cohort there was no statistically significant difference (HR 0.71, *p* = 0.387). However, in a small cohort of KRAS mutation positive patients the combination therapy achieved a significant prolongation of median PFS (HR 0.18, *p* = 0.006). The randomized, double-blind MARQUEE study investigated the addition of tivantinib to erlotinib in previously treated NSCLC patients. After a planned interim analysis the trial was stopped due to futility [94]. In a recent preclinical model, tivantinib efficacy has been shown to be independent of MET inhibition in NSCLC cell lines [95]. The ALK/ROS1 inhibitor crizotinib was originally developed as MET inhibitor [96]. Clinical activity of crizotinib in MET amplified patients has been demonstrated [97]. Other TKIs with activity against MET are currently investigated in clinical trials including cabozantinib and foretinib.

## 7. RET

RET (rearranged during transfection) is a receptor tyrosine kinase and a known oncogene in thyroid cancer where translocations as well as activating mutations have been detected [98,99]. In NSCLC RET translocations can be detected in about 1.5% of patients predominantly in younger, light or never smokers with adenocarcinoma histology and poorly differentiated tumors [100]. RET translocations seem to occur mutually exclusive with other known oncogenic driver mutations or translocations [101]. The most often detected fusion variant KIF5B-RET occurs through a paracentric inversion on chromosome 10 [14,102,103]. Additional gene fusion partners including CCD6, NCOA4 and TRIM33 have been described [104,105].

Alectinib is a potent inhibitor of ALK and has shown antitumor activity against RET positive NSCLC [106]. RET inhibition with vandetanib, sunitinib, and sorafenib resulted in loss of cell viability and abrogation of the transformed phenotype in preclinical models [14,102,103]. In a preliminary report of a phase II trial (NCT01639508) investigating the multi-tyrosine kinase inhibitor cabozantinib confirmed partial responses have been described in two of three RET-positive patients [104]. The third patient showed a prolonged stable disease. The activity of vandetanib for lung adenocarcinoma patients with RET translocation has been demonstrated in two case reports [107,108]. Clinical trials investigating a variety of different multi-kinase TKIs with activity against RET (e.g., vandetanib, cabozantinib, ponatinib) are currently ongoing.

## 8. PIK3CA

The phosphatidylinositol 3-kinase (PI3K)-AKT-mTOR pathway is one of the most often deregulated signaling cascades in human cancers, including NSCLC. PIK3CA encodes the catalytic subunit of PI3K. In NSCLC mutations and amplifications are detected in 2% and 12%–17%, respectively [109,110]. The incidence of PIK3CA mutations seems to be higher in lung squamous cell carcinoma [111]. PIK3CA mutations can occur in combination with other known driver mutations like EGFR or KRAS mutations as well as in the setting of acquired EGFR TKI resistance [112,113]. The fact that up to 70% of patients with a PIK3CA mutation harbor other coexisting mutations or rearrangements in other oncogenes supports the hypothesis that PIK3CA mutations are not oncogenic driver mutations per se in NSCLC [112]. Preclinical data suggest that tumors harboring PIK3CA mutations are highly sensitive to PI3K inhibitors [114]. Clinical trials with PI3K inhibitors as well as mTOR inhibitors are ongoing.

## 9. FGFR1

FGFR1 is a member of the FGFR family of receptor tyrosine kinases (FGFR1–4). FGFRs are transmembrane tyrosine kinases interfering with the RAS/RAF/MAPK and the PI3K/AKT signaling pathways. FGFR amplifications have been detected in about 20% of squamous cell carcinomas and in a lower frequency (about 5%) of adenocarcinomas [115,116]. FGFR1 amplification is more common in male smokers and associated with a poor outcome [117]. However, the negative prognostic impact of FGFR1 amplifications was not confirmed in a Caucasian patient cohort [118]. Clinical trials with FGFR inhibitors are currently ongoing. Activating mutations in the FGFR2/3 genes have recently been described in lung squamous cell carcinoma and have shown to be sensitive to FGFR inhibition [119].

## 10. DDR2

Discoidin domain receptor 2 (DDR2) together with DDR1 are receptor tyrosine kinases involved in cell adhesion, migration and proliferation [120]. In NSCLC DDR2 mutations have been described with a frequency of nearly 4% [121]. In a preclinical model the multikinase TKI dasatinib with activity against DDR2 showed activity in squamous carcinoma cell lines harboring DDR2 mutations [121]. Currently, a clinical trial with dasatinib in DDR2 mutant NSCLC is ongoing.

## 11. Other Genomic Aberrations

MEK1 (also named MAP2K1) is a serine-threonine kinase with mutations occurring in approximately 1% of NSCLC (mostly adenocarcinoma) [122]. NTRIK1 fusions have recently been described approximately 3% in never smokers with lung adenocarcinoma not harboring other oncogenic driver aberrations [123]. The NTRK1 gene encodes the high-affinity nerve growth factor receptor (TRKA protein). In a preclinical model two fusion variants (MPRIP-NTRK1, CD74-NTRK) have shown to have oncogenic potential [123]. RXDX-101 is an oral small molecule inhibitor of TrkA, TrkB and TrkC, as well as ROS1 and ALK. In a phase I trial RXDX-101 has demonstrated clinical activity in TRK-fusion positive lung cancer [124].

PTEN loss with subsequent AKT overexpression occurs in one third of NSCLC cases and is associated with poor prognosis [125]. PTEN mutations occur in 4%–8% of NSCLC and are more commonly detected in squamous cell histology and in patients with a smoking history [126]. Clinical trials investigating PI3K inhibitors in patients with PTEN deficiency are ongoing.

## 12. Conclusions

The therapeutic landscape of NSCLC therapy has profoundly changed since the first discovery of activating EGFR mutations and the development of specific EGFR TKIs. This has introduced the era of “personalized medicine”. There are still a growing number of new genomic aberrations in NSCLC serving as potential new predictive biomarkers and drug targets. Comprehensive genomic characterization of lung cancer tissue has recently been reported with high-quality [111,127,128]. In Western countries more than 50% of patients have at least one genomic aberration potentially amenable to specific therapeutic intervention [129]. Furthermore, the Lung Cancer Mutations Consortium has recently published their data showing that for patients harboring an actionable oncogenic driver treated with genotype-directed therapy the median survival is more than three years [7]. Although, this is a highly selected patient population it clearly shows that advances in the molecular understanding and novel treatment options during the last decade have significantly improved the outcome for a subgroup of NSCLC patients. However, as treating physicians and specialists in thoracic oncology we should not forget about the still larger subgroup of NSCLC patients not harboring an actionable genomic aberration. Beside putting our research focus on the detection of novel potentially predictive biomarkers, the investigation of new more specific and more potent inhibitors of specific signaling pathways further examination of potential resistance mechanisms in patients progressing during therapy with a targeted molecule and the investigation of novel treatment approaches with activity in the resistant setting are urgently needed.

Current international guidelines recommend analysis of the following seven genes before starting palliative intent therapy for lung adenocarcinoma: KRAS, EGFR, ALK, ROS1, HER2, BRAF, RET [130]. However, only for EGFR and ALK approved therapies are currently available. With the growing number of genomic drivers and novel molecularly targeted agents in incrementally smaller patient subgroups it’s a basic necessity to establish collaborative groups and foster preclinical and clinical research. Furthermore, novel approaches in clinical research have to be determined as the evaluation of targeted therapies can be highly challenging when the genomic aberrations are very rare. Recently, a novel trial design (so called “basket trials”) have been initiated. These trials include patients based on the presence of a predictive molecular marker independent of the tumor histology [131]. Recently, first results of the CUSTOM (Molecular Profiling and Targeted Therapies in Advanced Thoracic Malignancies) have been reported [132]. This trial was seeking to identify molecular biomarkers in advanced NSCLC, small-cell lung cancer, and thymic malignancies and to simultaneously evaluate five targeted therapies in patients grouped by molecular markers along with tumor histology. The five targeted therapies included erlotinib (EGFR mutations), the MEK inhibitor selumetinib (KRAS, HRAS, NRAS, and BRAF mutations), the AKT inhibitor MK2206 (PIK3CA, AKT1, and PTEN mutations), lapatinib (HER2 mutations), and sunitinib (KIT and PDGFRA mutations).

Another challenge with the growing number of targeted therapies is how to overcome the inevitable acquired resistance to these therapies. The concept of repeated biopsies at the time of tumor progression to investigate the specific resistance mechanism is nowadays an established approach and indispensable component of clinical trials. The optimal sequence for the use of multiple inhibitors, potential combinations of targeted therapeutics with each other, with chemotherapy or with immunotherapeutic approaches or the intercalation of these different treatment options as well as the tolerability of these approaches are currently investigated.

Although metastatic NSCLC is still an incurable disease, recent advantages in the understanding of the underlying molecular mechanisms, the implementation of high-throughput technologies including next-generation sequencing in the clinical diagnostic and the discovery of targeted therapeutics have clearly improved the prognosis and quality of life for a substantial group of patients with advanced NSCLC.
